# Supplementary material for: Effects of iron supplements and iron-containing micronutrient powders on the gut microbiome in Bangladeshi infants: a randomized controlled trial
Source: Nat Commun. 2024 Oct 5;15:8640. doi: 10.1038/s41467-024-53013-x (PMC11452624; doi:10.1038/s41467-024-53013-x)
Supplement: Supplementary file 1 — Supplementary Information [file 41467_2024_53013_MOESM1_ESM.pdf]

**16S rRNA V4 primers and PCR conditions**

| | |
|---|---|
| Primer 515F-OH1 | GTGACCTATGAACTCAGGAGTCGGACTACNVGGGTWTCTAAT |
| Primer 806R-OH2 | CTGAGACTTGCACATCGCAGCGTGYCAGCMGCCGCGGTAA |

PCR1: 94°C for 3 minutes; 20 cycles at 94°C for 45 seconds each; 55°C for 1 minute; 72°C for 90 seconds. Final step at 72°C for 10 minutes. Amplicons were diluted 1 in 10 before PCR2 (with reaction conditions as above, except an increase to 25 cycles.

**Table S1: Baseline Characteristics of participants in the BRISC microbiome sub-study (shotgun metagenomic samples only).**

| | Iron<br>N=99 | MNPs<br>N=109 | Placebo<br>N=111 |
|---|---|---|---|
| Union | | | |
| Bhulta | 28/99 (28.3%) | 32/109 (29.4%) | 40/111 (36.0%) |
| Golakandail | 30/99 (30.3%) | 35/109 (32.1%) | 38/111 (34.2%) |
| Rupganj | 41/99 (41.4%) | 42/109 (38.5%) | 33/111 (29.7%) |
| Household Food Secure status* | 81/99 (81.8%) | 82/106 (77.4%) | 88/109 (80.7%) |
| Age extra food in addition to breastfed (months) | 6.0 (5.0-6.0) | 6.0 (5.0-6.0) | 6.0 (5.0-6.0) |
| Hemoglobin concentration (g/L) venous, mean (SD) | 109.8 (10.7) | 110.9 (8.8) | 110.3 (9.8) |
| Anemia venous† | 42/99 (42.4%) | 45/105 (42.9%) | 49/110 (44.5%) |
| Ferritin (ug/L) | 24.6 (11.6-32.8) | 24.7 (15.4-46.7) | 24.8 (13.8-38.7) |
| Iron deficient‡ | 33/98 (33.7%) | 26/102 (25.5%) | 31/105 (29.5%) |
| Iron deficient anemia venous¶ | 23/98 (23.5%) | 15/102 (14.8%) | 24/105 (22.9%) |
| Hepcidin – (ng/mL), median (IQR) | 29.5 (19.0-51.7) | 31.2 (15.4-56.9) | 32.8 (18.0-57.2) |
| Low hepcidin (hepcidin <10 ng/mL) | 7/92 (7.6%) | 12/97 (12.4%) | 11/101 (10.9%) |
| C-reactive protein (mg/L) | 0.95 (0.38-2.75) | 0.80 (0.31-3.14) | 0.84 (0.34-3.20) |

| | Iron<br>N=99 | MNPs<br>N=109 | Placebo<br>N=111 |
|---|---|---|---|
| Inflammation-- | 14/98 (14.3%) | 18/102 (17.6%) | 18/105 (17.1%) |
| Length/height-for-age z-score | -1.31 (0.96) | -1.28 (1.02) | -1.25 (0.97) |
| Stunted‖ | 23/99 (23.2%) | 28/108 (25.9%) | 22/111 (19.8%) |
| Weight-for-age z-score | -0.55 (0.96) | -0.67 (1.06) | -0.46 (0.90) |
| Underweight** | 6/99 (6.1%) | 8/108 (7.4%) | 6/111 (5.4%) |
| Weight-for-length/height z-score | 0.34 (0.92) | 0.17 (1.01) | 0.42 (0.98) |
| Wasting†† | 1/99 (1.0%) | 2/108 (1.9%) | 1/111 (0.9%) |

Data are n/N (%) unless stated otherwise. MNPs = Micronutrient powders; SD = Standard Deviation; IQR = Interquartile range (25th to 75th percentile).
*Household food security was assessed and defined using the Household Food Insecurity Access Scale.
†Anaemia was defined as venous haemoglobin <110 g/L.[1]
‡Iron deficiency was defined as ferritin level <12 µg/L or <30 µg/L if C-reactive protein was >5 mg/L).[1]
¶Iron deficiency anaemia was defined as concurrent iron deficiency and anaemia.
--Inflammation was defined as C-reactive protein >5 mg/L.
‖Stunting was defined as length-for-age z-score <-2.[2]
**Underweight was defined as weight-for-age z-score <-2.[2]
††Wasting was defined as weight-for-length z-score <-2.[2]

**Figure S1: CONSORT Diagram of the BRISC Microbiome Sub-study.**

Figure S1 created with BioRender.com released under a Creative Commons Attribution-NonCommercial-NoDerivs 4.0 International license (https://creativecommons.org/licenses/by-nc-nd/4.0/deed.en).

# SUPPLEMENTARY FIGURE 1

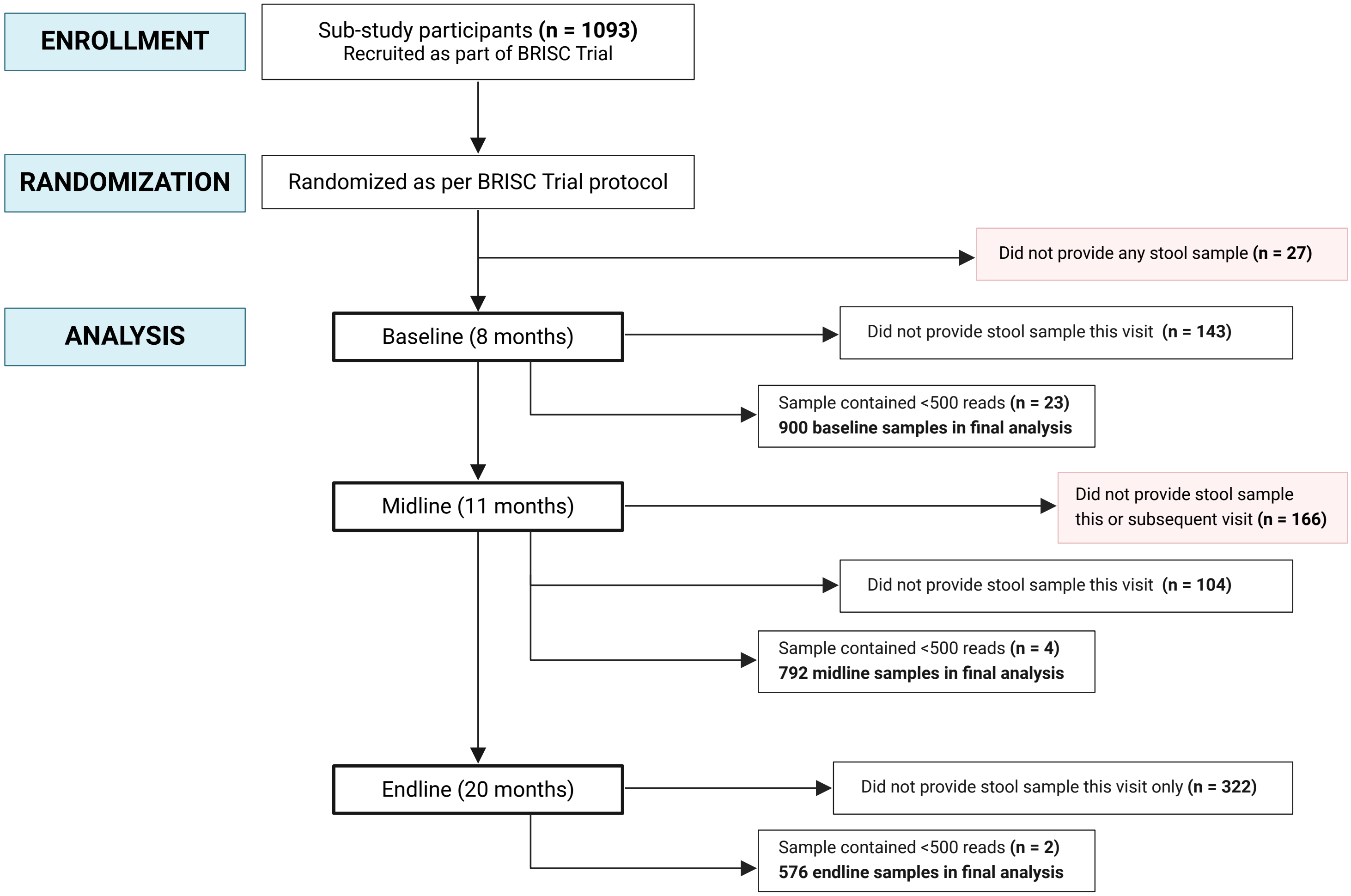

**Figure S2: Heatmap showing changes in abundance of genes relating to microbiome functional profile by sampling time point.**

Heatmap demonstrating relative differences in abundance of genes relating to microbiome functional profile by sampling time point across all trial arms, with baseline as reference. The 50 features exhibiting the greatest relative change in abundance (increase – in red – or decrease – in blue – from baseline) are listed. Abundance is expressed as $-\log_{10}$(FDR-adjusted p-value) * sign($\log_2$-fold change) as per the heatmap calculation used by *MaAsLin2*. (n = 255 at baseline, n = 231 at post-intervention and n = 313 at post-follow-up time points) (Shotgun metagenomic data). Source data for this figure are provided in the Source Data file.

# SUPPLEMENTARY FIGURE 2

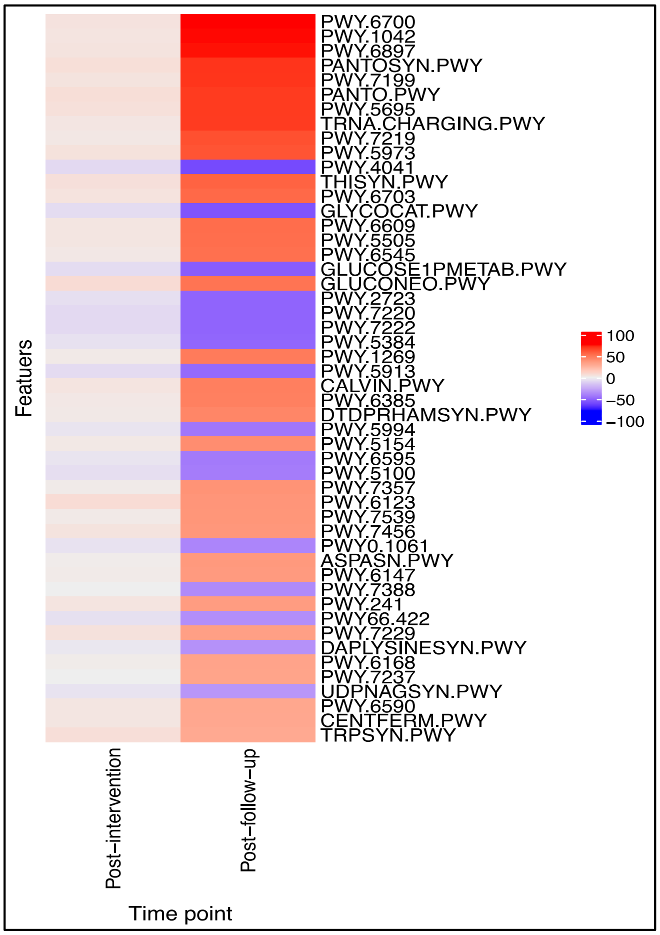


| Abbreviation | Pathway |
|---|---|
| PWY-6700 | Queuosine biosynthesis |
| PWY-1042 | Glycolysis IV (plant cytosol) |
| PWY-6897 | Thiamin salvage II |
| PANTOSYN-PWY | Pantothenate and coenzyme A biosynthesis I |
| PWY-7199 | Pyrimidine deoxyribonucleosides salvage |
| PANTO-PWY | Phosphopantothenate biosynthesis I |
| PWY-5695 | Urate biosynthesis/inosine 5-phosphate degradation |
| TRNA-CHARGING-PWY | tRNA charging |
| PWY-7219 | Adenosine ribonucleotides de novo biosynthesis |
| PWY-5973 | cis-vaccenate biosynthesis |
| PWY-4041 | γ-glutamyl cycle |
| THISYN-PWY | Superpathway of thiamin diphosphate biosynthesis I |
| PWY-6703 | preQ0 biosynthesis |
| GLYCOCAT-PWY | Glycogen degradation I (bacterial) |
| PWY-6609 | Adenine and adenosine salvage III |
| PWY-5505 | L-glutamate and L-glutamine biosynthesis |
| PWY-6545 | Pyrimidine deoxyribonucleotides de novo biosynthesis III |
| GLUCOSE1PMETAB-PWY | Glucose and glucose-1-phosphate degradation |
| GLUCONEO-PWY | Gluconeogenesis I |
| PWY-2723 | Trehalose degradation V |
| PWY-7220 | Adenosine deoxyribonucleotides de novo biosynthesis II |
| PWY-7222 | Guanosine deoxyribonucleotides de novo biosynthesis II |
| PWY-5384 | Sucrose degradation IV (sucrose phosphorylase) |
| PWY-1269 | CMP-3-deoxy-D-manno-octulosonate biosynthesis I |
| PWY-5913 | TCA cycle VI (obligate autotrophs) |
| CALVIN-PWY | Calvin-Benson-Bassham cycle |
| PWY-6385 | Peptidoglycan biosynthesis III (mycobacteria) |
| DTDPRHAMSYN-PWY | dTDP-L-rhamnose biosynthesis I |

| Abbreviation | Pathway |
|---|---|
| PWY-5994 | Palmitate biosynthesis I (animals and fungi) |
| PWY-5154 | L-arginine biosynthesis III (via N-acetyl-L-citrulline) |
| PWY-6595 | Superpathway of guanosine nucleotides degradation (plants) |
| PWY-5100 | Pyruvate fermentation to acetate and lactate II |
| PWY-7357 | Thiamin formation from pyrithiamine and oxythiamine (yeast) |
| PWY-6123 | Inosine-5-phosphate biosynthesis I |
| PWY-7539 | 6-hydroxymethyl-dihydropterin diphosphate biosynthesis III (Chlamydia) |
| PWY-7456 | Mannan degradation |
| PWY0-1061 | Superpathway of L-alanine biosynthesis |
| ASPASN-PWY | Superpathway of L-aspartate and L-asparagine biosynthesis |
| PWY-6147 | 6-hydroxymethyl-dihydropterin diphosphate biosynthesis I |
| PWY-7388 | Octanoyl-[acyl-carrier protein] biosynthesis (mitochondria, yeast) |
| PWY-241 | C4 photosynthetic carbon assimilation cycle, NADP-ME type |
| PWY66-422 | D-galactose degradation V (Leloir pathway) |
| PWY-7229 | Superpathway of adenosine nucleotides de novo biosynthesis I |
| DAPLYSINESYN-PWY | L-lysine biosynthesis I |
| PWY-6168 | Flavin biosynthesis III (fungi) |
| PWY-7237 | Myo-, chiro- and scillo-inositol degradation |
| UDPNAGSYN-PWY | UDP-N-acetyl-D-glucosamine biosynthesis I |
| PWY-6590 | Superpathway of Clostridium acetobutylicum acidogenic fermentation |
| CENTFERM-PWY | Pyruvate fermentation to butanoate |
| TRPSYN-PWY | L-tryptophan biosynthesis |

**Figure S3: Subgroup analysis of adherence to trial intervention by trial arm.**

**A**: Violin plot presenting taxonomic alpha diversity – measured by Shannon and inverse Simpson indices – grouped by trial arm and restricted to participants with ≥70% adherence to trial intervention (16S rRNA data). **B**. Volcano plot illustrating differential abundance at the genus level by trial arm of the same adherence subgroup as measured immediately post-intervention (16S rRNA data). **C**. Volcano plot illustrating differential abundance at the species level by trial arm of the same adherence subgroup as measured immediately post-intervention (Shotgun metagenomic data). **D**. Volcano plot illustrating differential abundance of functional pathways by trial arm of the same adherence subgroup as measured immediately post-intervention (Shotgun metagenomic data). For **B-D** $\log_2$-fold change is shown on the x-axis and the $-\log_{10}$(FDR-adjusted p-value) on the y-axis. The horizontal red line indicates an FDR-adjusted p-value 0·05. Source data for Figure S3B-D are provided in the Source Data file.

# SUPPLEMENTARY FIGURE 3

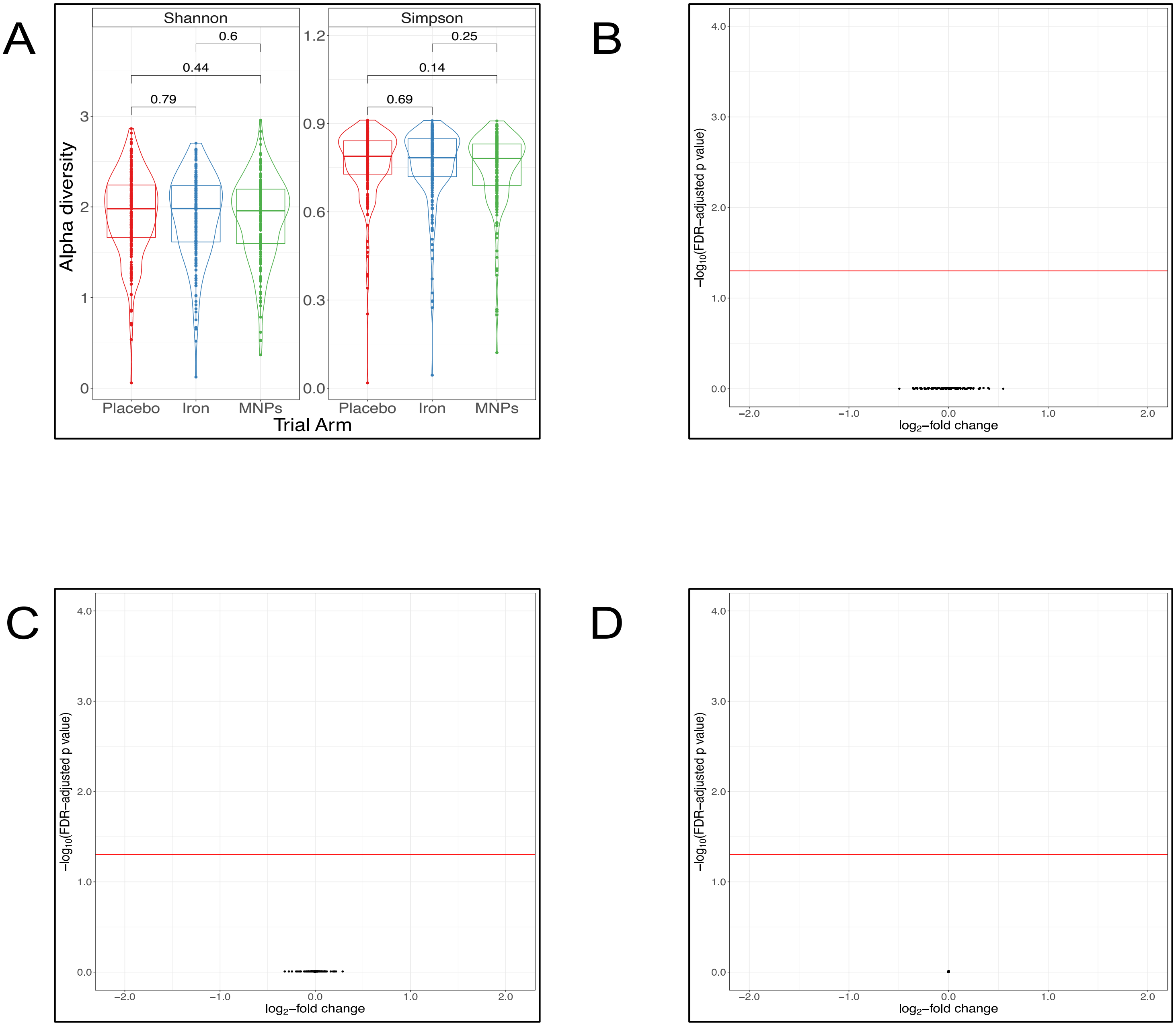

**Figure S4: Alpha diversity and differential abundance analysis by trial arm at the follow-up time point.**

**A**: Violin plot presenting taxonomic alpha diversity – measured by Shannon and inverse Simpson indices –by trial arm and measured at the post-follow-up time point (16S rRNA data). **B**. Volcano plot presenting differential abundance at the genus level by trial arm and measured at the post-follow-up time point (16S rRNA data). **C**. Volcano plot illustrating differential abundance at the species level by trial arm and measured at the post-follow-up time point (Shotgun metagenomic data). **D**. Volcano plot presenting differential abundance of functional pathways by trial arm and measured at the post-follow-up time point (Shotgun metagenomic data) (1. Pentose phosphate pathway (non-oxidative branch), 2. CMP-3-deoxy-D-manno-octulosonate biosynthesis I, 3. PWY.5154, 4. Thiamin salvage II, 5. Superpathway of L-aspartate and L-asparagine biosynthesis. 6. 6-hydroxymethyl-dihydropterin diphosphate biosynthesis III (Chlamydia), 7. Flavin biosynthesis I (bacteria and plants), 8. 6-hydroxymethyl-dihydropterin diphosphate biosynthesis I, 9. dTDP-L-rhamnose biosynthesis I, 10. L-isoleucine biosynthesis III, 11. Urate biosynthesis/inosine 5'-phosphate degradation, 12. Superpathway of branched amino acid biosynthesis). $Log_2$-fold change is shown on the x-axis and the $-log_{10}$(FDR-adjusted p-value) on the y-axis. The horizontal red line indicates an FDR-adjusted p-value 0·05. Source data for Figure S4B-D are provided in the Source Data file.

# SUPPLEMENTARY FIGURE 4

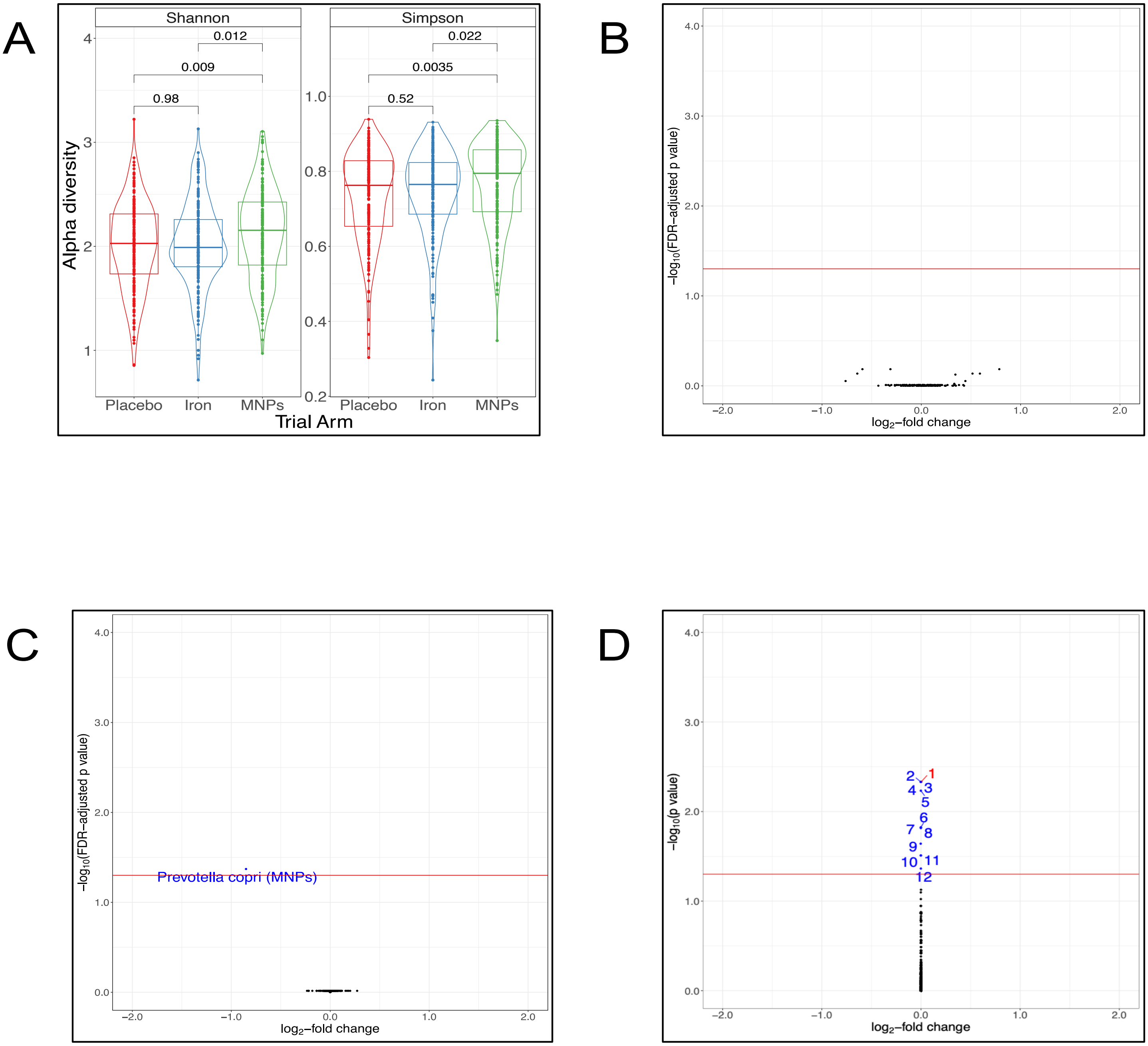

## Reference


1. World Health Organization. Iron deficiency anaemia: assessment, prevention and control — a guide for pro- gramme managers. (World Health Organization, Geneva, 2001).
2. de Onis, M., Onyango, A.W., Van den Broeck, J., Chumlea, W.C. & Martorell, R. Measurement and standardization protocols for anthropometry used in the construction of a new international growth reference. *Food Nutr Bull* **25**, S27-36 (2004).
